# Effect of a single session of ear acupuncture on pain intensity and postural control in individuals with chronic low back pain: a randomized controlled trial

**DOI:** 10.1590/bjpt-rbf.2014.0158

**Published:** 2016-06-16

**Authors:** Andrea Ushinohama, Bianca P. Cunha, Leonardo O. P. Costa, Ana M. F. Barela, Paulo B. de Freitas

**Affiliations:** 1 Laboratório de Análise do Movimento, Programa de Pós-graduação em Ciências do Movimento Humano, Universidade Cruzeiro do Sul, São Paulo, SP, Brazil; 2 Programa de Mestrado e Doutorado em Fisioterapia, Universidade Cidade de São Paulo (UNICID), São Paulo, SP, Brazil; 3 Musculoskeletal Division, The George Institute for Global Health, Sydney, Australia

**Keywords:** physical therapy, auriculotherapy, body balance, equilibrium, ultrasound

## Abstract

**Background:**

Ear Acupuncture (EA) is a form of acupuncture in which needles are applied to the external ear and has been used in multiple painful conditions. Low back pain (LBP) is highly prevalent in active individuals and causes high economic burden to health systems worldwide. LBP affects the person’s ability to keep balance, especially in challenging conditions.

**Objective:**

The aim of the study was to examine the effects of a single session of EA on pain intensity and body sway during postural tasks.

**Method:**

Eighty adults with LBP and pain intensity equal to or greater than 4 (0-10 scale) were randomly allocated (1:1) to EA group (EAG) or placebo group (PG). Initially, the level of pain intensity was assessed. Next, participants stood still on a force plate either with feet in parallel or in semi-tandem and with eyes open or closed. Then, the EAG was treated with EA for 20 min and the PG was treated with detuned ultrasound. After the treatment, pain intensity was assessed again and the postural test was repeated. Pain intensity was the primary outcome and center of pressure sway area and speed were the secondary outcomes measured.

**Results:**

Results revealed that pain intensity decreased in both groups after treatment, but decreased more in the EAG. For postural control, no effect of treatment and no interaction between treatment and postural condition on body sway were found.

**Conclusion:**

Those findings indicate that EA is better than placebo to reduce pain, but neither treatment has any effect on postural control.

## BULLET POINTS

- One session of ear acupuncture (EA) temporarily reduces pain in individuals with low back pain.
- Although EA reduces pain, body balance is not affected by EA.
- EA could be used to reduce pain and disability momentarily in individuals with low back pain.

## Introduction

Ear acupuncture (EA), also known as auriculotherapy, is a variant of the traditional acupuncture in which needles or beads are placed in specific points of the outer ear. The French version of EA is based on the assumption that the human body is represented in the outer ear as an inverted fetus and that there is a relationship between distinctive anatomical sites and specific points in the ear^1^. Previous studies showed that EA is effective to reduce pain as a single treatment^2^ ^-^ ^5^ or as an adjuvant of other types of treatment^6^ ^-^ ^9^. For instance, EA was better than sham EA to reduce pain in adolescents with dysmenorrhea^5^ and in older adults immediately after a hip fracture^3^. However, in most of EA studies, participants either received several sessions of EA or kept the needles inserted for hours or even days. To our knowledge, except for Barker and colleagues’, no study investigated the effect of a single session of EA on pain intensity^3^. It is important because, when compared to analgesic and anti-inflammatory drugs, EA has minimal side effects and is relatively inexpensive. Also, EA could be an alternative to acupuncture because the treated individual does not need to remain lying down or remove part of his/her clothing and it can be easily applied anywhere (e.g. at home, office, medical center).

Low back pain (LBP) is defined as pain or discomfort located below the costal margin and above the inferior gluteal folds^10^. LBP is highly prevalent and persistent and has become an economic burden to health systems and companies worldwide^11^ ^,^ ^12^. Studies have shown that LBP causes changes in sensorimotor function and, specifically, in the ability to control body balance and orientation^13^ ^,^ ^14^. Individuals with LBP increase their body sway when compared to individuals without pain^15^. It has been suggested that LBP disrupts the ability of the nervous system to obtain appropriate proprioceptive information from the muscles of the lumbar region^15^ and it could affect the accurateness of the information about the trunk position in space. However, the difference between LBP and healthy individuals in postural tasks is seen mainly in more demanding tasks^13^ ^,^ ^16^. In this study, we tested the hypothesis that a single application of EA in individuals with chronic LBP would be sufficient to temporarily reduce pain intensity and improve balance, reducing their body sway, mainly in more complex postural tasks, when compared to a placebo treatment. Balance improvement would occur because reduction in pain intensity would improve the quality of proprioceptive information about trunk position in space and, consequently, the postural control system could function properly to reduce body sway.

## Method

### Study type

We performed a two-arm, randomized, placebo-controlled trial with a blinded assessor. This trial was approved by the local Research Ethics Committee of Universidade Cruzeiro do Sul, São Paulo, SP, Brazil (approval number 142/2013) and was prospectively registered at^17^ (Trial registration number NCT01995279).

### Inclusion and exclusion criteria

To be included in our sample, male and female participants should be between 18 and 50 years-old, have complaints of non-specific chronic LBP (≥12 weeks), and report a minimum of 4 on a pain rating scale of 0 to 10 at the moment of the assessment. The cut-off of 4 was chosen to allow only participants with some room for improvement, as patients with very low levels of pain tend not to respond to any therapy^18^. Individuals were excluded if they reported other musculoskeletal or neurological conditions. Individuals who underwent spine surgery or had complaints of dizziness were excluded from the sample. Furthermore, participants should not be seeking treatment to reduce LBP and should not have taken painkillers and anti-inflammatory medicine 24 hours prior to the test.

### Source of the participants

Participants were recruited in São Paulo, SP, Brazil, by personal invitation by the researchers to individuals they knew to have LBP. Some individuals responded to flyers fixed in points close to the data collection sites. They were evaluated at three different places: at the Motion Analysis Lab of the Universidade Cruzeiro do Sul, at a fitness center; and at a cookie factory between December 2013 and February 2014. At all places, two quiet rooms were used, one for evaluation of primary and secondary outcomes and another for treatment.

### Experimental procedure

The participants were informed about the study procedures and signed the informed consent form. Next, the assessor recorded the participants’ demographic and anthropometric data and asked them to rate their back pain on a scale of 0 to 10^19^ ^,^ ^20^. After, the participants answered the Brazilian-Portuguese version of the Roland-Morris Disability Questionnaire (RMDQ) to assess their daily life disability associated with LBP^21^ ^-^ ^23^.

Finally, the participants’ postural control was assessed. They were asked to stand barefoot as still as possible on a portable force platform (Kistler, 9286A), with their arms beside the trunk. They were tested in two visual conditions (eyes open and closed) and in two base of support conditions: parallel feet (standing in a comfortable position with feet side-by-side and hip-width apart) and semi-tandem stance (hallux of the rear foot touching the calcaneus bone of the front foot). Therefore, the participants’ balance was assessed in four conditions: parallel feet with eyes open (PFEO); parallel feet with eyes closed (PFEC); semi-tandem with eyes open (STEO); and semi-tandem with eyes closed (STEC). During the eyes-open conditions, participants were asked to fix their gaze on a target placed in front of them at eye level, and at a distance of 1 meter. In the eyes-closed conditions, they were instructed to close their eyes and maintain the same position as in the eyes-open conditions. Three trials were performed per condition. Each trial lasted 35 s and their order was randomized and determined by drawing.

After the postural assessment, each participant was taken to another room where a therapist immediately opened the sealed opaque envelope with the kind of treatment the participant would receive. After the treatment, the participant went back to the first room and performed the post-treatment evaluation.

### Participant allocation and interventions

Participant allocation was randomized in a 1:1 ratio using a specific website^24^ by a person not involved in the study. The randomization codes were placed by this person in consecutively numbered, sealed, and opaque envelopes ensuring concealed allocation into two groups. Eighty participants were allocated into one of two groups.

### Ear acupuncture

**Figure 1:**
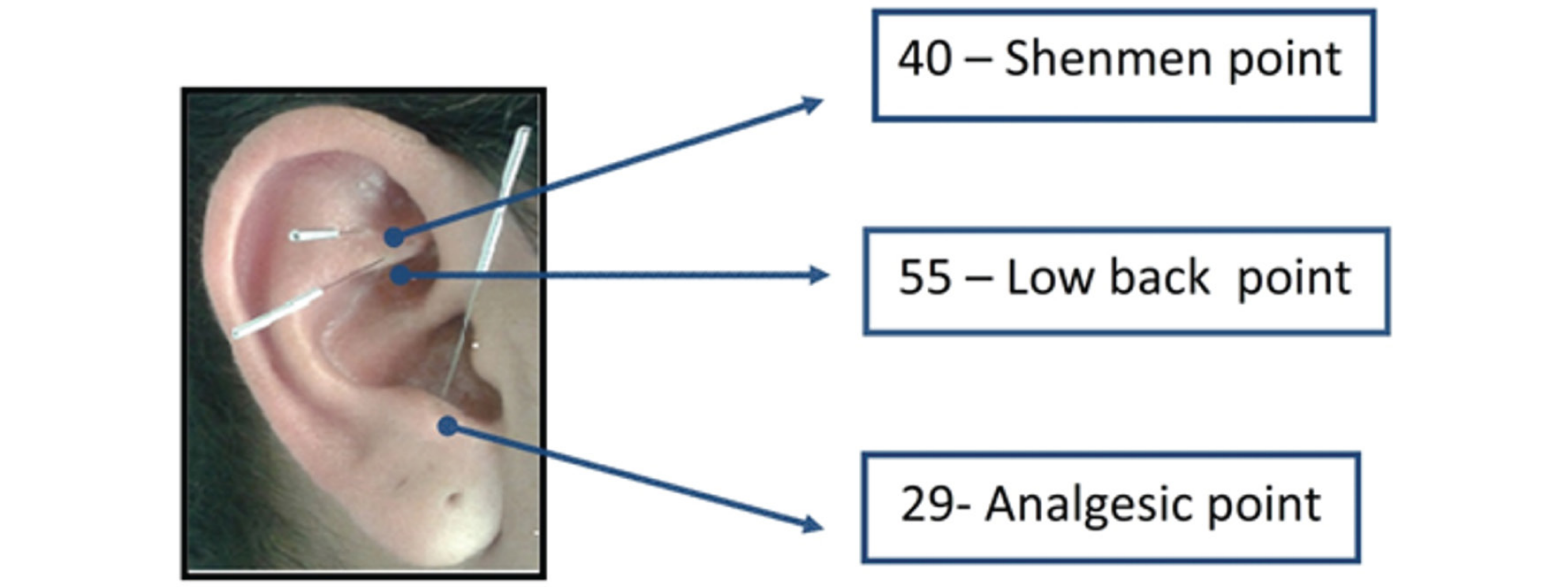
Points in the ear where needles were applied to reduce low back pain^4^ ^,^ ^25^.

The first group (EA group) received EA in three points: point 29 (analgesic point), point 40 (shenmen point), and point 55 (low back point) (Figure 1). These points were selected because they are commonly used in individuals with LBP^4^ ^,^ ^25^. The needles used in this procedure were disposable Dong Bang needles (0.15×30 mm). The needle application was performed by an experienced therapist. This therapist had 11 years of experience using EA as treatment for LBP.

### Placebo

The second group (placebo) received application of detuned ultrasound (Sonopulse III, Ibramed, Brazil). The ultrasound machine was turned on, but not activated (i.e. no vibration was transmitted to the skin). The head of the ultrasound was placed in light contact with the skin of the painful lower back region and was kept in constant circular motion for minimal interference with the painful area. The placebo therapy was applied by the same therapist. Both sessions lasted 20 minutes, during which the participants remained lying in a therapy bed. The detuned ultrasound treatment is commonly used as placebo treatment in control trials and shows high level of credibility^26^.

### Blinding

The assessor was blinded to the participants’ group allocation. This “blindness” was assessed as the assessor wrote down on each participant’s data sheet her opinion about what kind of treatment the participant received and her opinions were compared with the randomization codes after the end of the study^27^.

### Outcomes

The primary outcome of the study was the pain intensity which was assessed by the numeric pain rating scale (NPRS). This is an 11-point numeric pain scale, ranging from 0 to 10, with 0 meaning “no pain” and 10 meaning “unbearable pain”.

Two secondary outcomes were obtained from the balance test. Force signals were recorded at 200 Hz by a customized LabView routine (National Instruments, USA), digitally filtered by a low-pass, 4^th^ order, zero-lag, Butterworth filter with a 10 Hz cut-off frequency and used to calculate the center of pressure (COP), which is the point of application of the resultant of vertical forces acting on the surface of support^28^. The changes in the COP position over time are directly related to the body sway^29^. COP trajectories were calculated in the anterior-posterior (AP) and medial-lateral (ML) direction. After that, the first and final 2.5 s of the COP AP and ML time-series were removed and only the central 30 s were used for the outcomes’ calculation.

The secondary outcomes were COP sway area (SA) and COP sway speed (SS). SA estimates the dispersion of the COP trajectory by using principal component analysis (PCA). In short, PCA computes two axes of the ellipse based upon the COP signal dispersion. This ellipse contains 85% of the COP data and its area is considered the COP SA^29^. COP SS is simply the path travelled by the COP divided by the time used in data processing. Sway path length is calculated by summation of the planar distance between two consecutive COP positions^29^.

### Sample size

The sample size calculation was performed using the primary outcome (i.e. NPRS) according to the following criteria: difference between groups equal to 1 point; standard deviation of the difference equal to 1.5 points; power of 80%; alpha of 5%; and possible sample loss of 10%. These estimates for sample size calculation were derived from previous trials^30^ ^,^ ^31^. The result revealed the need for 40 participants in each group. We are aware that the minimum clinically important difference in patients with LBP is 2 points. However, a sample size calculation using 2 points instead of 1 would suggest a very small sample size, which would cause high statistical imprecision. Consequently, we decided to estimate our sample using 1 point.

### Statistical analyses

The statistical analyses started with normality tests. The Shapiro-Wilk test showed that none of the outcomes showed normal distribution. For pain intensity, after we have unsuccessfully tried data transformation, we decided to run a non-parametric test. In order to test differences between treatments, we calculated the difference in pain intensity (DPI) subtracting the individuals’ NPRS reported at baseline, which was not different between groups, by the NPRS reported after the treatment (DPI=NPRS_pre_–NPRS_post_). After that, we performed a Mann-Whitney U test for this variable. For COP SA and SS, we successfully transformed the data and both variables became normally distributed. COP SA was transformed by logarithm function [log_10_(SA)] while COP SS was transformed by its inverse (1/SS). Next, we performed a multivariate analysis of variance (MANOVA) to test the effect of treatment (EA and placebo), period (pre-treatment and post-treatment), and postural condition (PFEO, PFEC, STEO, and STEC) on transformed SA and SS values. The factors period and condition were treated as repeated measures. Univariate analyses and post-hoc tests were used when necessary. The alpha level was set at .05 and Bonferroni corrections were employed when needed. For DPI, the effect size was calculated from the Z value obtained from the Mann-Whitney U test as [r_ES_=(z/√N)], with r_ES_ higher than 0.5 being interpreted as a large effect, higher than 0.3 being interpreted as medium effect, and higher than 0.1 interpreted as small effect according to Cohen’s standards^32^. For SA and SS, effect size information was provided by values of partial eta square (η^2^)^32^. All statistical analyses were performed using IBM SPSS (version 19), which was performed on an intention-to-treat basis (i.e. we analyzed patients in the groups to which they were originally randomly assigned).

## Results

**Figure 2:**
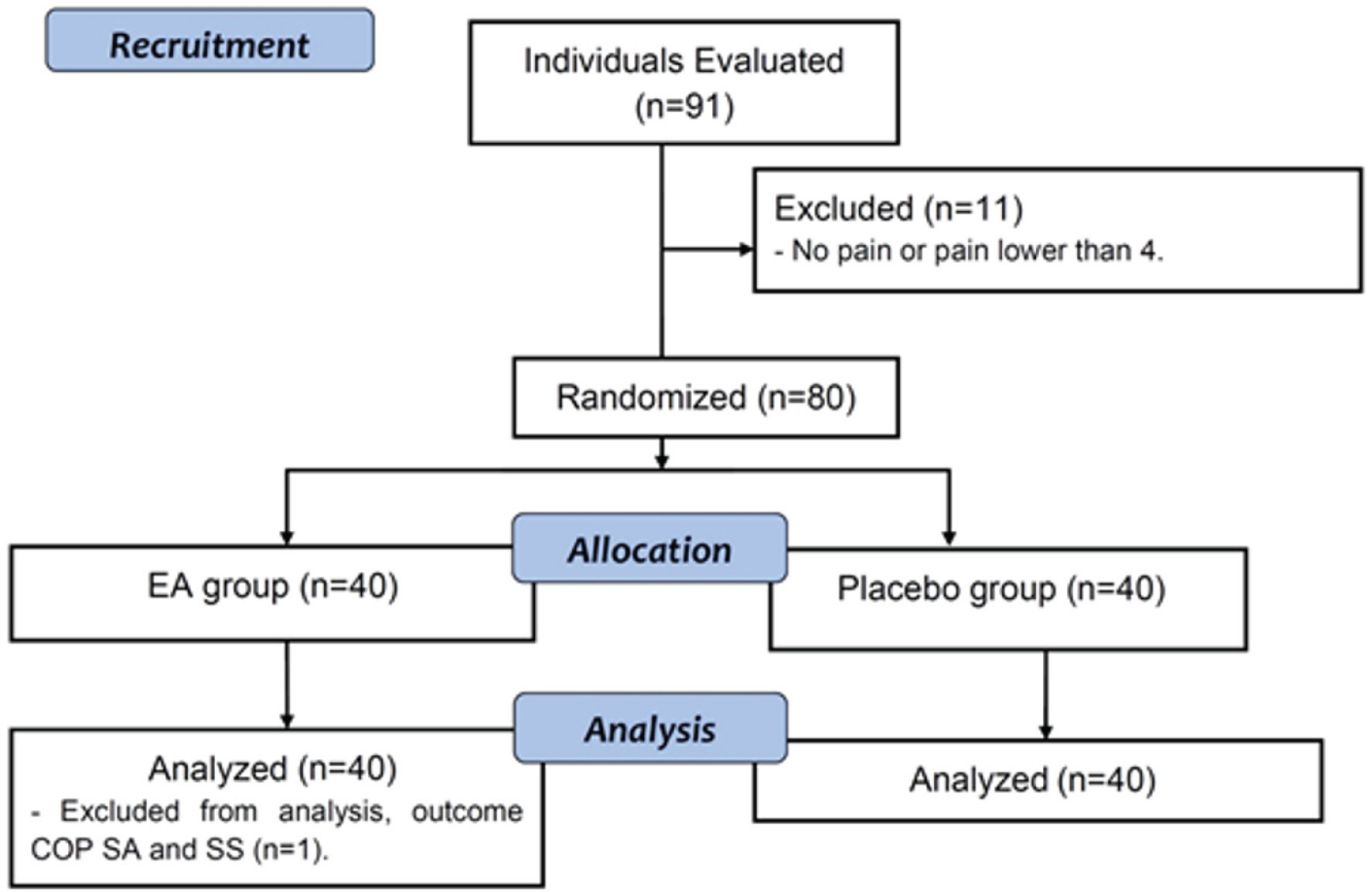
Study flow diagram.

Ninety-one individuals were contacted and came to the testing sites. Eleven did not report pain equal to or greater than 4 and were not tested. Thus, 80 participants were randomly assigned to one of the two groups (Figure 2). Most of them were female (62.5%), 35 years old on average, and had a relatively long presence of symptoms (around 43 months). The pain intensity reported at the beginning of this trial was moderate (5.7). However, the participants presented low level of disability as shown by the RMDQ (4.2 points from 0 to 24) (Table 1). All participants were assessed for pain intensity and one participant from the EA group was excluded from the balance test after the treatment because she felt dizzy and was unable to perform the tasks. This participant was analyzed on an intention-to-treat basis for these secondary outcomes by imputing the data from baseline (last value carried forward). As this participant reported her level of pain after the intervention, no imputation technique was required. It is unclear whether this dizziness was related to the treatment or not. No other adverse event occurred.

**Table 1:** Participants’ individual characteristics. Except for sex, data are presented as mean and standard deviation.

**Characteristics**	**Groups**	
	**EA**	**Placebo**
**Sex**		
Female	27	23
Male	13	17
**Age (years-old)**	37.9 (7.7)	34.3 (8.9)
**Body mass (kg)**	72.4 (17.7)	69.7 (14.4)
**Body height (m)**	1.66 (0.08)	1.67 (0.08)
**Symptom duration (months)**	46.3 (38.3)	39.5 (35.4)
**Disability (RMDQ 0-24)**	4.5 (3.6)	4.1 (3.02)

### Assessor blinding

The assessor was correct about the treatment received in 56.25% (p=.25, chi-square test) of the cases, indicating that the blinding of the assessor was successful.

### Primary outcome: pain intensity

The median (interquartile range) of the DPI from post to pre-treatment of the EA group was 2 (4) and the placebo group was 1 (4). The Mann-Whitney U test revealed that the absolute DPI was higher in the EA group as compared to the placebo group (U=611.5, Z=1.857 p=.032, one-tailed, r_ES_=.21), meaning that the reduction in pain was larger in the EA group than in the placebo one, with a small effect size according to Cohen's standards^32^.

### Secondary outcomes: COP sway area and speed

MANOVA revealed no effect of treatment (Wilks’ Lambda=.937, F(2,77)=2.58, p>.05, η^2^=.08) and no interaction between treatment and period (Wilks’ Lambda=.969, F(2,77)=1.24, p>.05, η^2^=.03), treatment and condition (Wilks’ Lambda=.958, F(6,73)=0.54, p>.05, η^2^=.04), and across treatment, period, and condition (Wilks’ Lambda=.936, F(6,73)=0.83, p>.05, η^2^=.06). However, MANOVA revealed effect of period (Wilks’ Lambda=.738, F(2,77)=13.67, p<.001, η^2^=.26) and condition (Wilks’ Lambda=.038, F(6,73.)=308.8, p<.001, η^2^=.96) and interaction between period and condition (Wilks’ Lambda=.799, F(6,73)=3.07, p<.05, η^2^=.2). Univariate analysis revealed that the interaction between period and condition was observed only for SS (p<.005). Tests of simple effect revealed that SS was lower in the post- than in pre-treatment in PFEO, PFEC, and STEC, but not in STEO. Moreover, univariate analysis revealed that the main effect of period was found in SS (F(1,78)=27.44, p<.001, η^2^=.26), but not in SA (F(1,78)=2.33, p>.05, η^2^=.03). Finally, univariate analysis revealed effect of condition on both variables (SA: F(3,234)=459.7, p<.001, η^2^=.86 | SS: F(3,234.)=617.3, p<.001, η^2^=.96). Post-hoc tests revealed that all conditions were different from each other. Specifically, SA and SS increased from the simplest (PFEO) to the most complex condition (STEC) (Table 2).

**Table 2:** COP sway area (upper panels) and sway speed (lower panels) averaged across participants for both groups (ear acupuncture – EA – and placebo) during four postural conditions (PFEO: parallel feet and eyes open; PFEC: parallel feet and eyes closed; STEO: semi-tandem and eyes open; STEC semi-tandem and eyes closed). Numbers within parentheses indicate standard deviation.

	**Group**		**PFEO**	**PFEC**	**STEO**	**STEC**
Sway Area (mm^2^)	EA	**Pre**	71.05 (43.05)	98.48 (52.42)	199.90 (98.60)	375.62 (170.22)
		**Post**	70.40 (40.54)	97.98 (61.61)	177.58 (85.81)	323.70 (137.34)
	Placebo	**Pre**	85.50 (59.06)	130.56 (104.22)	213.12 (96.98)	364.15 (149.29)
		**Post**	84.46 (45.91)	126.39 (86.58)	199.21 (91.08)	384.20 (137.89)
Sway Speed (mm/s)	**Group**		**PFEO**	**PFEC**	**STEO**	**STEC**
	EA	**Pre**	7.83 (1.26)	9.77 (1.91)	17.5 (3.18)	26.91 (7.24)
		**Post**	7.57 (1.42)	9.21 (2.35)	17.11 (3.19)	25.02 (6.14)
	Placebo	**Pre**	8.77 (2.24)	11.8 (4.65)	18.58 (3.29)	28.36 (6.37)
		**Post**	8.43 (2.01)	10.64 (2.84)	18.05 (3.95)	27.8 (6.92)

## Discussion

We tested the hypothesis that a single session of EA would be enough to temporarily reduce pain and improve balance in individuals with LBP. The results partially confirmed the hypothesis. While a single session of EA was effective to momentarily reduce pain intensity, EA did not improve body balance.

EA has been used by clinicians to reduce pain in different health conditions^3^ ^-^ ^9^. Our results showed that a single session of EA was better than the placebo treatment to temporarily reduce pain in individuals with LBP. It corroborates the results of Barker et al.^3^, who investigated the effect of the application of EA on older adults who had just suffered a hip fracture and were being taken to the hospital in an ambulance. Older adults who received EA felt less pain and anxiety and presented lower heart rate than the ones who received sham EA^3^. A few studies have already investigated the effect of EA on individuals with LBP. For instance, Yeh et al.^4^ found that participants with chronic LBP who received EA felt approximately 70% less pain than the ones who received sham EA after one month of treatment. In addition, Sator-Katzenschlager et al.^25^ found that the treatment with EA was successful as an adjuvant of a pharmacological treatment to reduce pain. Unlike other studies, our study assessed the efficacy of EA in reducing pain temporarily after a single session. Our results showed that the application of EA could be beneficial even if someone is treated just once. The reduction in pain intensity shows that the application of EA has an immediate, albeit small, effect and could be considered as a non-pharmacological alternative for these patients, especially given that no adverse effects are observed after application. Furthermore, patients with a lower level of pain after EA may be more likely to improve with exercises for pain and disability in patients with chronic LBP. However, more trials with high methodological quality are needed to confirm the results of this study.

We also examined the functional result of an intervention with EA. It is known that LBP negatively influences balance and this effect is more evident in more complex tasks^13^. However, our findings showed no effect of treatment on postural control. Thus, the reduction in pain intensity was not enough to cause changes in postural control. A likely explanation could be that what affects the balance system is not the current pain intensity, but the altered postural control system as a result of prolonged pain. Findings from previous studies revealed a direct association between existing pain intensity and postural control^33^. The presence of pain caused by the continuous discharge of the nociceptors located at the lumbar region would reduce the activation of proprioceptors, mainly the muscle spindles, thus affecting balance^15^. However, our results do not confirm this hypothesis given that, despite the reduction in pain intensity, postural control remained unaffected after the treatment. A second possibility is that LBP would affect the organization and excitability of cortical and subcortical areas related to postural control^34^. Consequently, reduction in current pain intensity would not affect postural control at the moment of the reduction. Our results appear to support this suggestion. Another possible explanation is that the participants of this study presented low scores in the RMDQ, which could be indicative of the severity of LBP. In a systematic review, Mazaheri et al.^13^ associated the severity of LBP with changes in body balance. Therefore, the lack of treatment effect on postural control could be due to the level of severity of LBP in the individuals investigated in this study and we are currently investigating this possibility.

To our knowledge, this is the first study to assess the effect of a single session of EA on pain intensity in individuals with chronic LBP. In addition, the effect of EA on postural control has yet to be investigated.

This is a low risk of bias trial with proper randomization procedure, participant allocation, adequate sample size, and assessor blinding, which can be considered as an asset. On the other hand, our study has some limitations that need attention. First, we recruited only individuals who were not seeking treatment, which could explain the low scores in the RMDQ and subsequent lack of influence of the treatment on body balance. Thus, a new trial could be conducted to assess individuals with greater disability due to LBP. Secondly, our study only assessed the immediate effects of EA treatment, and different results (i.e. stronger effect size in pain intensity and significant improvement in body balance) may have been observed if more treatment sessions had been provided. Thirdly, although most textbooks and references on EA advocate the same protocol used in this study, we acknowledge that there are other possibilities for EA. Therefore, our results are only generalizable for this type of treatment protocol. Finally, we did not monitor the level of credibility of the treatments and did not use a real placebo EA group (i.e. Sham EA) in this study. Both monitoring the level of credibility of the treatments and using a sham EA group would have strengthened our findings and provided additional support for the use of EA for temporary pain reduction in individuals with LBP.

## Conclusion

In conclusion, the findings showed that EA is effective in temporarily reducing pain intensity, but this is not enough to improve body balance. This knowledge is important because physicians and therapists could suggest the use of EA to reduce acute pain momentarily in individuals with LBP in any condition, namely for individuals who are unable to take traditional painkillers.
